# A metastasis biomarker (MetaSite *Breast*™ Score) is associated with distant recurrence in hormone receptor-positive, HER2-negative early-stage breast cancer

**DOI:** 10.1038/s41523-017-0043-5

**Published:** 2017-11-08

**Authors:** Joseph A. Sparano, Robert Gray, Maja H. Oktay, David Entenberg, Thomas Rohan, Xiaonan Xue, Michael Donovan, Michael Peterson, Anthony Shuber, Douglas A. Hamilton, Timothy D’Alfonso, Lori J. Goldstein, Frank Gertler, Nancy E. Davidson, John Condeelis, Joan Jones

**Affiliations:** 1 Montefiore Medical Center, Albert Einstein College of Medicine, 1695 Eastchester Road, 10461 Bronx, NY USA; 2 ECOG-ACRIN Research Group, Boston, MA USA; 3 Albert Einstein College of Medicine, Bronx, NY USA; 4 Mt. Sinai School of Medicine, New York, NY USA; 5 MetaStat, Inc, Boston, MA USA; 6 Weill Cornell Medicine, New York, NY USA; 7 Fox Chase Cancer Center, Philadelphia, PA USA; 8 Massachusetts Institute of Technology, Boston, MA USA; 9 University of Pittsburgh Cancer Institute, Pittsburgh, PA USA

## Abstract

Metastasis is the primary cause of death in early-stage breast cancer. We evaluated the association between a metastasis biomarker, which we call “Tumor Microenviroment of Metastasis” (TMEM), and risk of recurrence. TMEM are microanatomic structures where invasive tumor cells are in direct contact with endothelial cells and macrophages, and which serve as intravasation sites for tumor cells into the circulation. We evaluated primary tumors from 600 patients with Stage I–III breast cancer treated with adjuvant chemotherapy in trial E2197 (NCT00003519), plus endocrine therapy for hormone receptor (HR)+ disease. TMEM were identified and enumerated using an analytically validated, fully automated digital pathology/image analysis method (MetaSite *Breast*™), hereafter referred to as MetaSite Score (MS). The objectives were to determine the association between MS and distant relapse free interval (DRFI) and relapse free interval (RFI). MS was not associated with tumor size or nodal status, and correlated poorly with Oncotype DX Recurrence Score (*r* = 0.29) in 297 patients with HR+/HER2- disease. Proportional hazards models revealed a significant positive association between continuous MS and DRFI (*p* = 0.001) and RFI (*p* = 0.00006) in HR+/HER2- disease in years 0–5, and by MS tertiles for DRFI (*p* = 0.04) and RFI (*p* = 0.01), but not after year 5 or in triple negative or HER2+ disease. Multivariate models in HR+/HER- disease including continuous MS, clinical covariates, and categorical Recurrence Score (<18, 18–30, > 30) showed MS is an independent predictor for 5-year RFI (*p* = 0.05). MetaSite Score provides prognostic information for early recurrence complementary to clinicopathologic features and Recurrence Score.

## Diagnostics: Digital pathology test predicts relapse risk

A new diagnostic assay helps predict risk of metastasis among women with a particular form of early-stage breast cancer. A team led by Joseph Sparano from the Albert Einstein College of Medicine in the Bronx, New York, evaluated primary tumors from 600 women who were treated with chemotherapy and endocrine therapy for stage I–III breast cancer that was hormone receptor-positive (HR+). They used a digital image analysis test called MetaSite *Breast*, which measures the microscopic anatomical structure of the tumor microenvironment. The patients’ scores on MetaSite *Breast* did not correlate with tumor size, nodal status or genetic signature, but it did serve as a reliable predictor of five-year disease recurrence among women with HR+ cancer who also tested negative for human epidermal growth factor receptor 2. The test could offer patients valuable prognostic information.

## Introduction

Metastasis is the primary cause of death in breast cancer, the most frequently diagnosed cancer and the leading cause of cancer death among women, accounting for 25% of the total cancer cases (1.68 million) and 15% of the cancer deaths (520,000) worldwide.^1,2^ Although breast cancer mortality rates have declined due to screening and more effective adjuvant systemic therapies,^3^ more accurately identifying metastatic risk in order to minimize overtreatment or undertreatment, and developing new approaches to prevent metastasis remain major clinical chalenges.^4^ Elucidating the biology underpinning the metastatic process offers one potential approach to addressing these challenges.

Studies evaluating breast cancer cell dissemination at single cell resolution using novel multiphoton imaging tools and mouse models have shown that tumor cells stream toward blood vessels.^5^ Collection of streaming tumor cells using an in vivo invasion assay for gene expression profiling has led to the identification of the Invasion Signature and discovery of pathways that are upregulated in streaming tumor cells with transendothelial migration activity,^6–15^ which includes differential expression of invasive Mena isoforms that regulate cancer cell streaming, transendothelial migration, and ultimately intravasation of breast tumor cells.^16–21^ A subpopulation of Mena-expressing comes into direct contact with endothelial cells and macrophages, forming microanatomic structures that we have called “Tumor MicroEnvironment of Metastasis”, or “TMEM”, which serve as a doorway for other streaming tumor cells to intravasate.^22,23^ TMEM are found in primary human breast cancers, as well as regional lymph node and distant metastases.^24^

TMEM structures may be identified in formalin-fixed paraffin-embedded human breast cancers by a triple immunostain identifying where macrophages (anti-CD68), endothelial cells (anti-CD31), and streaming tumor cells (anti-panMena) are in direct contact.^25,26^ In an initial proof-of-concept case:control study of 60 patients with early breast cancer, median TMEM Score was significantly higher in primary tumors in cases with distant recurrence compared with controls without distant recurrence (median TMEM density 150 vs. 50, *p* = 0.00006).^25^ Moreover, in a case–control study of 259 case–control pairs nested in a large population-based study of early-stage breast cancer patients, TMEM Score was positively associated with risk of distant recurrence in estrogen receptor (ER) + , HER2- disease when evaluated in multivariate model including tumor size, nodal status, grade and IHC4 Score (odds ratio 2.67, *p* = 0.004 for highest vs. lowest tertile), and did not correlate with tumor size, nodal status, grade, or IHC4.^26^ In another report, TMEM Score did not correlate Oncotype Recurrence Score.^24^

Based on this initial evidence indicating a strong association between TMEM Score and distant metastasis in ER+, HER2-negative breast cancer, we undertook another study to confirm the association between TMEM Score and recurrence in this breast cancer subtype, and to explore the association in triple negative and HER2+ breast cancer. We used a prospective-retrospective study design that utilized biospecimens from a well annotated and uniformly treated clinical trial cohort, an approach that provides a high level of evidence supporting the clinical validity of a biomarker.^27^ The study population included 600 patients with high-risk stage I–III breast cancer and 0–3 positive axillary nodes who all received standard chemotherapy, plus endocrine therapy if the tumor was hormone receptor (HR) positive.^28^ In a subset of 297 patients with HR+/HER2- breast cancer, we also evaluated risk of metastasis in association with TMEM Score in joint models including the Oncotype DX Recurrence Score, a widely used multiparameter gene expression assay that has shown clinical validity and utility in this population,^29^ and in a prospectively conducted clinical trial.^30^ Moreover, we used a fully automated and scalable clinical assay for identification and enumeration of TMEM (MetaSite *Breast*™) utilizing digital pathology methods coupled with image analysis, which demonstrated high analytical accuracy, reproducibility, and precision,^31^ and hence use the term MetaSite Score to describe the assay results in this report.

## Results

### MetaSite Score in overall cohort and by breast cancer subtype

The characteristics of the study cohort, overall and by breast cancer subtype, is shown in Table 1. The MetaSite Score in all 600 cases ranged from of 0 to 199, and the weighted mean MetaSite Score was 19.9. The weighted mean MetaSite Score was significantly lower in the HR+/HER2- subtype than in the triple negative subtype (16.1 vs. 23.8, *p* = 0.001) or the HER2+ subtype (16.1 vs. 26.2, *p* = 0.003), while the difference between triple negative and HER2+ was not significant (23.8 vs. 26.2, *p* = 0.59). As expected, virtually all patients with triple negative disease (99.5%) and most with HER2+ disease (69%) had a high Recurrence Score of >30 because low ER and high HER2 expression contribute to a higher Recurrence Score.^29^

**Table 1:** Distribution of clinicopathologic factors and mean weighted Metasite Scores by breast cancer subtype

	Triple negative		HR+/HER2-		HER2+		Total	
	# (*n* = 200)	Weighted %	# (*n* = 297)	Weighted %	# (*n* = 103)	Weighted %	# (*n* = 600)	Weighted %
Age (years)								
<=40	42	22.7%	33	10.8%	14	13.2%	89	14.4%
41–50	77	40.6%	96	32.9%	39	39.7%	212	36.1%
51–60	53	24.1%	102	35.6%	30	28.4%	185	31.3%
>60	28	12.6%	66	20.7%	20	18.8%	114	18.2%
Premenopausal	107	56.4%	129	43.3%	46	45.8%	282	47.3%
Central ER/PR IHC								
ER-positive	0	0%	264	90.5%	57	65.7%	321	61.7%
PR-positive	0	0%	273	91.8%	47	54.8%	320	60.7%
Tumor size								
<=2 cm	83	44.0%	157	54.4%	44	45.4%	284	50.1%
2.1 to 5.0 cm	108	52.1%	131	42.1%	53	50.6%	292	46.2%
>5 cm	9	3.9%	9	3.5%	6	4.0%	24	3.7%
Axillary nodal status								
Node negative	148	80.9%	145	54.3%	63	67.9%	356	63.8%
1–3 positive nodes	52	19.1%	152	45.7%	40	32.1%	244	36.2%
Central grade								
Low	2	1.1%	77	27.7%	6	8.0%	85	17.2%
Intermediate	18	9.0%	141	48.8%	28	30.9%	187	35.0%
High	180	89.9%	79	23.5%	69	61.1%	328	47.8%
Recurrence Score								
<18	0	0%	153	54.1%	13	17.7%	166	33.3%
18–30	1	0.5%	94	31.5%	10	13.3%	105	20.0%
>30	199	99.5%	50	14.4%	80	69.0%	329	46.7%
Treatment arm								
AT	89	48.8%	170	51.4%	51	47.2%	310	50.0%
AC	111	51.2%	127	48.6%	52	(58.8%)	290	50.0%
MetaSite Score								
Weighted mean	–	23.8	–	16.1	–	26.2	–	19.9

ER/PR/HER2 classification: 2/600 cases did not have central immunohistochemistry (IHC) available, but local IHC and gene expression results were concordant for these two cases, so they were included and classified based on those results (one case HR+/HER2- and one case TN). ER/PR was classified as positive for Allred Score of 3 or higher as described in ref. 49 HER2 expression was classified in a central lab as positive if 3 + by IHC or FISH amplification by 2007 ASC0-CAP guidelines^48^

*AT* doxorubicin/docetaxel, *AC* doxorubicin/cyclophosphamide

### Clinicopathologic features by empirical MetaSite tertile group

The empirical tertile cutpoints for the entire cohort in the weighted distribution were 0 to 5 (190 cases, weighted percent 33.3%), 6 to 17 (196 cases, weighted percent 33.5%) and 18 to 199 (214 cases, weighted percent 33.2%). The characteristics of patients in each MetaSite Score empirical tertile group are shown in Table 2. There were significant differences in MetaSite Score tertile distribution (high, intermediate, low MetaSite Score) for age 40 or younger (37% vs. 31% vs. 21%, *p* = 0.02) and older than 60 years (34% vs. 31% vs. 49%, *p* = 0.04), ER (48%, vs. 70% vs 67%, *p* < 0.001) and PR (49% vs. 65% vs. 68%, *p* < 0.001) expression, and any grade (6% vs. 18% vs. 62% for low grade, *p* < 0.001; 20% vs. 40% vs. 44% for intermediate grade, *p* < 0.001; 73% vs. 42% vs. 28% for high grade, *p* < 0.001), but not tumor size or axillary nodal metastases. These findings are consistent with the observation of significantly higher MetaSite Scores in the triple negative and HER2+ compared with HR+/HER2- breast cancer.

**Table 2:** Distribution of clinicopathologic factors by empirical tertile of Metasite Score

	MetaSite Score		MetaSite Score		MetaSite Score		
	0–5		6–17		18–199		
	# (*n* = 190)	Weighted %	# (*n* = 196)	Weighted %	# (*n* = 214)	Weighted %	*p*-value
Age (years)							
<=40	21	9.3%	31	15.0%	37	19.0%	0.02
41–50	56	31.2%	78	40.6%	78	36.4%	0.21
51–60	64	34.8%	56	29.3%	65	29.8%	0.51
>60	49	24.6%	31	15.1%	34	14.8%	0.04
Premenopausal	75	40.6%	100	51.2%	107	50.0%	0.11
Central ER/PR IHC							
ER-positive	114	67.3%	118	69.5%	89	48.2%	<0.001
PR-positive	115	68.0%	111	65.1%	94	48.9%	<0.001
Tumor size							
<=2 cm	98	53.8%	91	49.1%	95	47.3%	0.45
2.1 to 5.0 cm	83	42.7%	98	46.9%	111	49.1%	0.47
>5 cm	9	3.5%	7	4.1%	8	3.6%	0.96
Axillary nodal status							
Node negative	111	61.8%	115	62.0%	130	67.6%	0.35
Central grade							
Low	46	27.4%	29	17.8%	10	6.3%	<0.001
Intermediate	77	44.2%	72	40.3%	38	20.3%	<0.001
High	67	28.4%	95	41.8%	166	73.4%	<0.001

*p*-values are for comparison of the weighted proportion in the three MetaSite groups for each row

### Relationship between MetaSite Score and Recurrence or Survival

The proportional hazards model including only MetaSite Score as a continuous covariate and the time-varying coefficient model that adds the variable time*MetaSite Score was fit overall and for each of the three breast cancer subtypes. Although there was no significant association between MetaSite Score and distant recurrence overall, there was strong evidence of an effect in the time-varying coefficient model in the HR+/HER2- group (*p* = 0.0002, two degree-of-freedom overall test for association), and the hazards were not proportional over time (*p* = 0.01) (Supplemental Table 1). A nearly significant association was also noted between MetaSite Score and breast cancer specific survival (*p* = 0.06) but not overall survival (OS) (*p* = 0.21) in the HR+/HER2- group in the time-varying coefficient model, which may be explained by the high proportion of deaths in this group that were not due to breast cancer (48/85 [56%]). The association between MetaSite Score and breast cancer death was not significantly time-dependent in the time-varying coefficient model (*p* = 0.54), but was significantly associated with breast cancer death in the proportional hazards model not adjusted for time (*p* = 0.04).

**Fig. 1:**
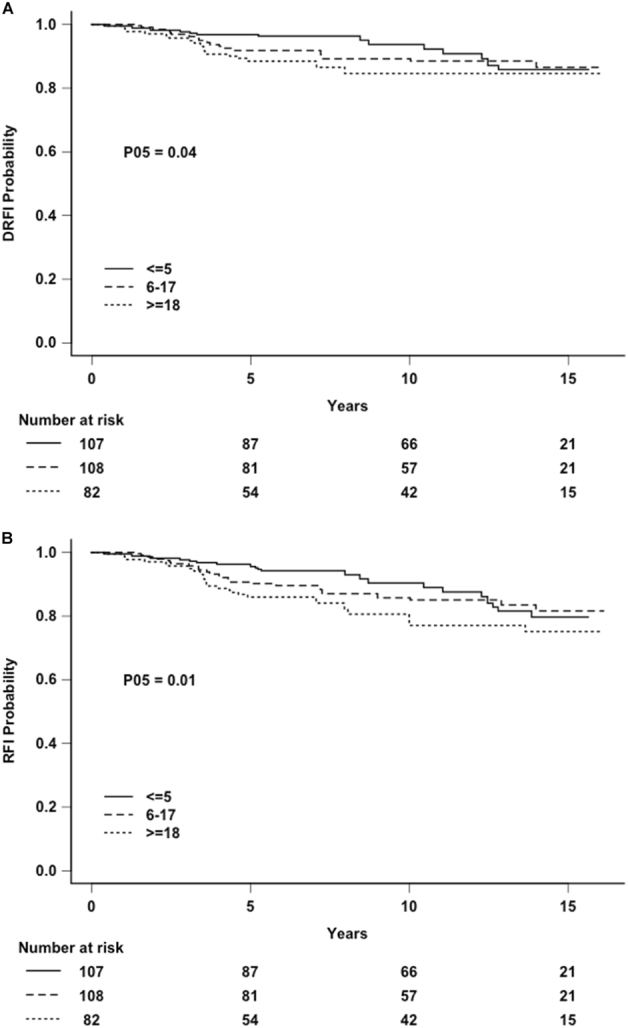
**a** Distant relapse free interval (DRFI) by MetaSite Score empirical tertile groups in hormone receptor-positive, HER2-negative disease (P05: *p*-value computed truncating follow-up at 5 years). **b** Relapse free interval (RFI) by MetaSite Score empirical tertile groups, in hormone receptor-positive, HER2-negative disease (P05: *p*-value computed truncating follow-up at 5 years)

In order to further evaluate the time-dependent effect for distant recurrence, the association between MetaSite Score and recurrence was evaluated using follow-up time intervals 0–5 years and 5–10 years, with no adjustment for other factors. Shown in Table 3 are the *p*-values and estimated coefficients for association of continuous MetaSite Score and risk of recurrence. A strong association was seen in the HR+/HER2- subtype in the 0–5 year period for distant relapse free interval (DRFI) (*p* = 0.001) and relapse free interval (RFI) (*P* = 0.00006), but not triple negative or HER2 + disease. The estimated coefficient was negative (and statistically non-significant) in all of the subsets in the 5–10 year period, suggesting that any effects are limited to the earlier time period. Kaplan–Meier analysis revealed a significant association between MetaSite Score and DRFI (*p* = 0.04) and RFI (*p* = 0.01) in the HR+/HER2- group when evaluated by empirical tertiles (</ = 5, 6–17, > / = 18) for the entire cohort with follow-up truncated at 5 years (Fig. 1a–b).

**Table 3:** Estimated association between continuous Metasite Score and risk of Distant Recurrence or any Recurrence

		0–5 Years		5–10 Years		Time by MetaSite *p*-value
Group	Endpoint	Estimated coefficient^a^	*p*-value	Estimated coefficient^a^	*p*-value	
Overall	DRFI	0.0062	0.10	−0.017	0.23	0.11
	RFI	0.0084	0.005	−0.0061	0.45	0.09
Triple negative	DRFI	−0.0081	0.34	–	–	–
	RFI	−0.0049	0.48	–	–	–
HR+/HER2-	DRFI	0.014	0.001	−0.0022	0.86	0.21
	RFI	0.015	0.00006	−0.0062	0.56	0.05
HER2+	DRFI	−0.0022	0.81	−0.082	0.16	0.18
	RFI	0.0040	0.53	−0.081	0.11	0.09

^a^ Estimated coefficient is the slope of the MetaSite Score variable in the proportional hazards model, which is the log hazard ratio for a 1-unit increase in the MetaSite Score value

### Relationship between Recurrence Score and Recurrence, and between MetaSite Score and Recurrence Score

In the 297 patients with HR+/HER2- disease, Recurrence Score was prognostic at 10 years when using classical cutpoints (<18, 18–30, or > 30) for DRFI (*p* = 0.0003) and RFI (*p* = 0.0004), and also when using TAILORx cutpoints (<11, 11–25, > 25) for DRFI (*p* = 0.007) and RFI (*p* = 0.003), although there was somewhat better separation among the low, intermediate, and high-risk categories using the latter definition (Supplemental Fig. 1A–D); results were similar if analysis was truncated at 5 years of follow-up (data not shown).

**Fig. 2:**
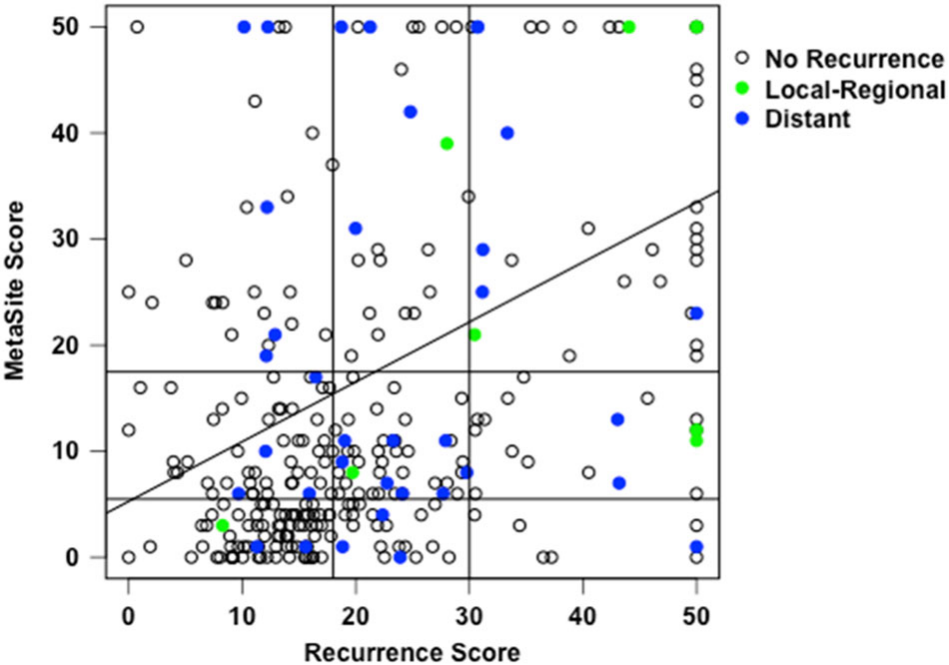
Correlation between MetaSite Score and Recurrence Score with both MetaSite Score and Recurrence Score (raw Pearson correlation *r* = 0.29) truncated at 50 (with all scores above 50 shown as 50). Recurrences occurring within 5 years are shown in colors, including all recurrences (RFI) and distant recurrences (DRFI)

The scatter plots of MetaSite Score by Recurrence Score are given in Fig. 2, showing poor correlation. The raw Pearson correlation between the continuous MetaSite Score and Recurrence Score is 0.27 in the overall sample and 0.29 in the HR+/HER2- subtype. Although MetaSite Score and Recurrence Score are both prognostic for recurrence in this cohort, they are only weakly correlated with each other. Figure 2 also shows individuals who had a recurrence or distant recurrence within the first 5 years, which accounted for the majority or recurrences (43 of 70 [61%]) and distant recurrences (35 of 49 [71%]) in the HR+/HER2- cohort (Supplemental Table 2). The figure illustrates a wide range of MetaSite scores within each Recurrence Score group, including the mid-range Recurrence Scores, however defined.

### Multivariate models in HR+, HER2- breast cancer subtype

Because the association between MetaSite Score and recurrence was time-dependent, proportional hazards models for DRFI and RFI in the HR+/HER2- subset using follow-up through 5 years were fit (Table 4). Clinical covariates including axillary nodal status (negative vs. positive), tumor size (</=2 cm vs. >2 cm) and grade (high vs. intermediate vs. low) were included in all models. MetaSite Score (continuous linear variable) showed a significant association with RFI (*p* = 0.04) and borderline association with DRFI (*p* = 0.08). If Recurrence Score (continuous linear variable) was included in the model instead of MetaSite Score, it was not significant for RFI (*p* = 0.51) or for DRFI (*p* = 0.90). If categorical Recurrence Score (<18, 18–30, >30) was added to the model including MetaSite Score (continuous linear variable) and clinical covariates, there remained significant association with RFI (*p* = 0.05) and a borderline association with DRFI (*p* = 0.10). If categorical rather than continuous MetaSite Score was used in the model, the association was not significant.

**Table 4:** Log hazard ratios (LHR), standard errors (SE) and *p*-values (P) from multi-factor models for DRFI and RFI using follow-up through 5 years

	DRFI		RFI	
	LHR (SE)	*p*	LHR (SE)	*p*
Model 1:				
Node Pos vs. Neg	0.43 (0.26)	0.10	0.42 (0.22)	0.06
Tumor size<=2 cm vs. >2	0.60 (0.37)	0.11	0.40 (0.33)	0.23
Grade Mod vs. Well	0.64 (0.54)	0.31	0.56 (0.50)	0.13
Poor vs. Well	0.96 (0.64)		1.12 (0.56)	
Continuous MetaSite Score	0.011 (0.006)	0.08	0.011 (0.005)	0.04
Model 2:				
Node Pos vs. Neg	0.39 (0.26)	0.14	0.40 (0.22)	0.07
Tumor size<=2 cm vs. >2	0.62 (0.37)	0.10	0.40 (0.33)	0.23
Grade Mod vs. Well	0.70 (0.54)	0.21	0.59 (0.50)	0.13
Poor vs. Well	1.30 (0.74)		1.30 (0.64)	
Continuous Recurrence Score	0.002 (0.017)	0.90	0.009 (0.014)	0.51
Model 3:				
Node Pos vs. Neg	0.54 (0.28)	0.05	0.52 (0.23)	0.03
Tumor size<=2 cm vs. >2	0.55 (0.38)	0.15	0.35 (0.34)	0.30
Grade Mod vs. Well	0.44 (0.55)	0.71	0.35 (0.51)	0.67
Poor vs. Well	0.55 (0.80)		0.61 (0.70)	
RS 18–30 vs. <18	0.85 (0.49)	0.18	0.91 (0.45)	0.13
> 30 vs. <18	0.57 (0.79)		0.79 (0.67)	
Continuous MetaSite Score	0.010 (0.006)	0.10	0.010 (0.005)	0.05

The three models differ only in the modeling of MetaSite Score and Recurrence Score. Model 1 includes only continuous MetaSite Score, Model 2 only continuous Recurrence Score, and Model 3 includes both categorical Recurrence Score. *p*-values for Grade and Categorical Recurrence Score are for any differences among the three categories

### Prognosis associated with MetaSite Score by Recurrence Score category

**Fig. 3:**
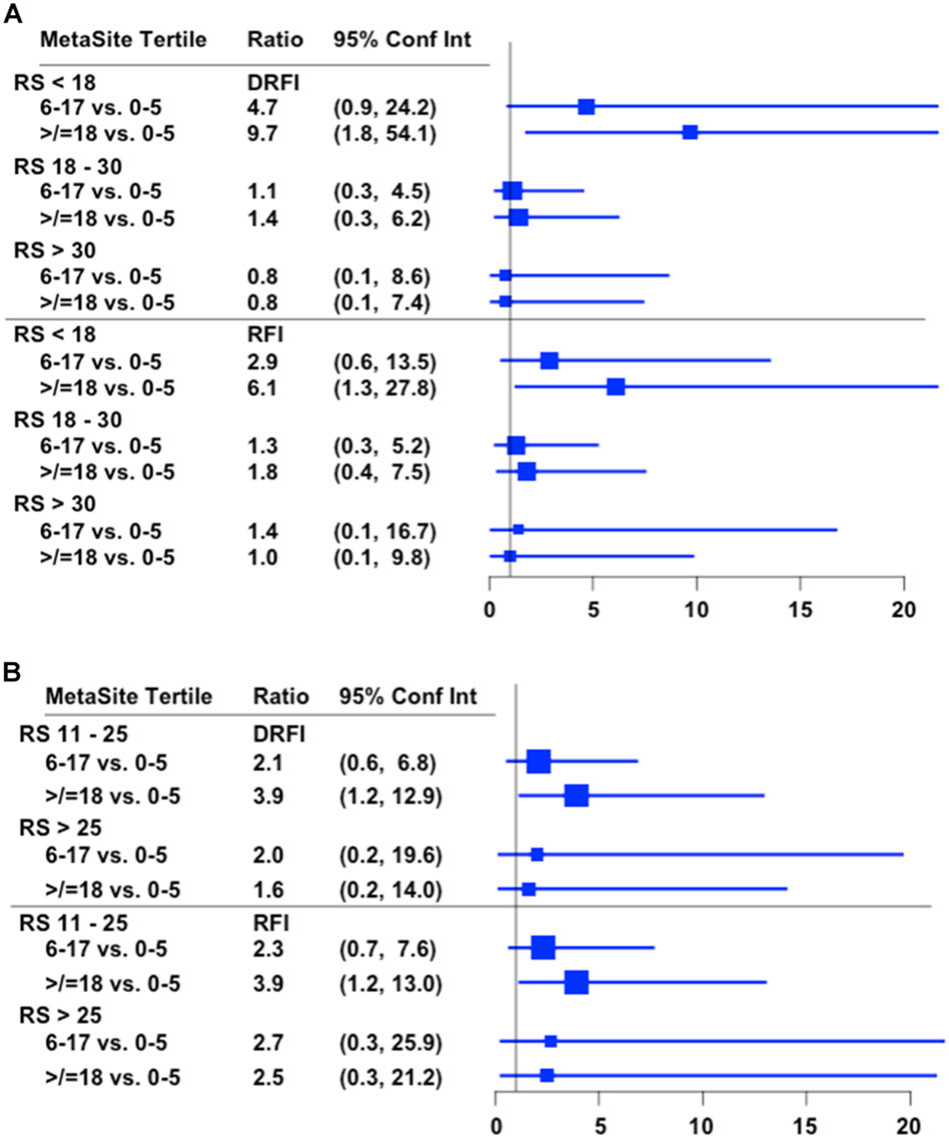
Hazard ratio for recurrence (and 95% confidence intervals) for MetaSite Score by empirical tertile group by categorical Recurrence Score, including classical definitions (3a) and TAILORx definitions (3b)

The complementary prognostic information provided by MetaSite Score was further examined by evaluating the hazard ratio for recurrence by Recurrence Score categories of low, intermediate and high risk, using both the classical (Fig. 3a) and TAILORx definitions in the HR+/HER2- group (Fig. 3b). Higher MetaSite Score (upper tertile vs. lower tertile) was associated with a 9.7-fold higher risk of distant recurrence (95% confidence intervals [CI] 1.8, 54.1) and 6.1-fold higher risk of overall recurrence (95% CI 1.3, 27.8) if the Recurrence Score was <18, but did not provide additional information for the intermediate or high Recurrence Score groups using the classical cutpoints (Fig. 3a). If the TAILORx cutpoints were used, there were too few recurrences to evaluate the contribution of MetaSite Score, although higher MetaSite Score was associated with a 3.9-fold higher risk of distant recurrence (95% CI 1.2, 12.9) in the group that had a mid-range Recurrence Score of 11–25 (Fig. 3b).

## Discussion

Using high-resolution two-photon microscopy in the MMTV-PyMT mammary carcinoma and patient-derived xenograft models, we have shown that: (1) transient blood vessel permeability induced by vascular endothelial growth factor (VEGF) and accompanying tumor cell intravasation occur exclusively at TMEM sites, (2) TMEM-associated macrophages are a subpopulation of tumor-associated macrophages that are pro-angiogenic (TIE2^Hi^/VEGF^Hi^), and (3) secretion of VEGF from TMEM macrophages leads to TMEM-associated transient blood vessel permeability, tumor cell intravasation, and an increase in circulating tumor cells.^23^ Prior proof-of-concept studies indicated that TMEM structures could be identified in human breast cancer using a triple immunohistochemical stain for Mena over-expressing tumor cells, macrophages, and endothelial cells all in direct contact,^25^ and that identification and enumeration of TMEM structures by manual methods was prognostic for distant recurrence in HR+/HER2- breast cancer independent of classical clinicopathologic features using primary tumor samples derived from a population-based cohort.^26^ Here, we report a second prospective-retrospective biomarker study confirming the clinical validity of the association between TMEM Score and distant recurrence, specifically early recurrence within 5 years of diagnosis, now in a uniformly treated clinical trial cohort of patients with HR+/HER2- early-stage breast cancer using an automated, high-throughput analytically validated assay (MetaSite *Breast*™) in a Clinical Laboratory Improvement Amendments (CLIA)-certified clinical diagnostic laboratory; hence, we use the term MetaSite Score to describe the assay and results here. Although MetaSite Score was not prognostic in triple negative or HER2+ breast cancer in this cohort, it is noteworthy that the scores were significantly higher in this high-risk subtypes compared with HR+/HER2-negative disease, which is consistent with the hypothesis that higher TMEM Score is associated with greater distant recurrence risk. Potential explanations for lack of prognostic association in poor risk breast cancer subtypes include the relatively small sample size and limited statistical power for these subtypes, and a threshold effect below which TMEM Score is prognostic, and above which higher TMEM Score is not associated with higher recurrence risk.

Another objective of this study was to evaluate whether MetaSite Score provided clinically useful prognostic information beyond that provided by Recurrence Score. Here, we show for the first time that MetaSite Score correlates poorly with Recurrence Score and provides complementary prognostic information for early recurrence, including patients with a mid-range Recurrence Score for whom uncertainty still exists about the benefit of chemotherapy.^4^ Three trials are currently evaluating the role of chemotherapy in patients with a mid-range Recurrence Score, including TAILORx (NCT00310180) in node-negative disease and an Recurrence Score of 11–25, and the RxPONDER (NCT01272037)^32^ and OPTIMA (ISRCTN42400492) trials in patients with up to nine positive axillary nodes and low to mid-range Recurrence Score (</=25).^33,34^

Because TMEM are believed to serve as the doorway for hematogenous dissemination and distant metastasis, the primary study endpoint in this analysis was distant recurrence as a first event, which accounted for 70% of the recurrences; the remaining 30% were localregional recurrences without concurrent distant recurrence as a first event. A limitation of our study was that distant recurrence occurring after localregional recurrence was not recorded, thus potentially underestimating the proportion of patients who eventually developed a distant recurrence. Isolated localregional recurrence without concurrent distant recurrence is known to be associated with a high risk of subsequent distant recurrence and breast cancer mortality.^35^ Thus, although there was only a borderline association between MetaSite Score and distant recurrence in the multivariate models including clinical covariates and categorical Recurrence Score, the significant association between MetaSite Score and all recurrences supports the robustness of the prognostic information provided by MetaSite Score.

In addition to the Recurrence Score assay, several other multiparameter gene expression assays are commercially available (e.g., MammaPrint^®TM^, Prosigna™, Breast Cancer Index℠).^36^ Although these signatures include different genes, they provide similar prognostic information that is driven largely by proliferation and estrogen-dependent genes and not by the intrinsic propensity of tumor cells to metastasize or interact with their microenvironment.^36–39^ Although direct comparison of the prognostic classification provided by some of these gene expression assays in the same cohort showed comparable prognostic information, they often provide discordant recurrence risk classification.^33^ Since MetaSite Score correlates poorly with Recurrence Score, captures different biologic information, and provides complementary prognostic information, it offers the potential to more accurately determine prognosis when added to multiparameter gene expression assays.

The tertiles of MetaSite Scores in the entire study population study were comparable in this clinical trial cohort using a fully automated assay in a clinical diagnostic laboratory (</=5, 6–17, >/=18) to the prior population-based cohort using a manual assay in an academic research laboratory (</=6, 7–22, >/=23);^26^ an analytic validation study of the MetaSite *Breast*™ assay using samples from the latter cohort yielded similar results.^31^ A new finding of the current study was that MetaSite Score was prognostic for distant recurrence within 5 years of diagnosis, but not after, which was not observed in our prior study in a population-based cohort.^26^ In E2197, all patients received four cycles of adjuvant doxorubicin plus cyclophosphamide (AC) or docetaxel (AT) (which were comparably effective^28^), whereas only 43% of patients in the population-based cohort received adjuvant chemotherapy. Since adjuvant chemotherapy (including a more prolonged course of sequential anthracycline-taxane therapy) prevents mainly early recurrence within the first 5 years, and sequentially administered regimens of longer duration are generally more effective than shorter regimens,^40^ a biomarker prognostic for early recurrence could be predictive for benefit from adjuvant chemotherapy, or more prolonged course of sequential anthracycline-taxane therapy. Moreover, early recurrence is associated with more aggressive disease and shorter survival than later recurrence,^41^ providing additional evidence that a biomarker prognostic for early recurrence provides clinically meaningful information. Additional studies in other cohorts will be required to determine the optimal MetaSite Score cutpoints for prognosis, and for prediction of chemotherapy benefit. Furthermore, studies are ongoing that are evaluating whether an a multiparameter biomarker that integrates TMEM Score with assessment of invasive Mena isoforms^42,43^ improves the accuracy of the prognostic information provided by the MetaSite Score alone.

Although we have focused on MetaSite Score as a prognostic biomarker in this study, and also described its potential for predicting chemotherapy benefit, it may also serve as a biomarker for agents targeting tumor-stromal interactions. For example, the TIE2 receptor is highly expressed in the TMEM-associated TIE2^Hi^/VEGF^Hi^ macrophage, and is responsible for the local release of VEGF from TMEM-associated macrophages causing vascular permeability specifically at TMEM sites.^23^ This offers the potential to use TMEM Score and other biomarkers reflecting TMEM function as predictive biomarkers for therapies targeting the Tie2-VEGF axis; a clinical trial testing this strategy for the first time is currently in progress (NCT02824575).

In conclusion, we report the results of a second prospective-retrospective validation study demonstrating the clinical validity of MetaSite Score in patients with ER+/HER2- breast cancer and show for the first time that it provides complementary prognostic information to Recurrence Score, specifically for early recurrence within 5 years of diagnosis. In both the original population-based cohort^26^ and the current clinical trial cohort, we used a high-throughput analytically validated assay (MetaSite *Breast*™) in a CLIA-certified clinical diagnostic laboratory for identification and enumeration of TMEM. Additional studies will be required to define the clinical utility of this assay in clinical practice.

## Methods

### Study population and treatment

The study utilized tumor specimens and clinical information from patients enrolled on trial E2197 (ClinicalTrials.gov identifier NCT00003519), coordinated by the Eastern Cooperative Oncology Group (ECOG), details of which have been reported.^28^ Briefly, 2952 eligible patients (of whom 2603 consented to future research) were randomly assigned to receive four 3-week cycles of doxorubicin 60 mg/m^2^ and cyclophosphamide 600 mg/m^2^ (AC) or docetaxel 60 mg/m^2^ (AT). Methods for selection of 776 cases (enriched for all relapsing cases) in the cohort have been previously described (including the 600 patients included in this analysis), and summarized in Supplemental Fig. 2.^44–47^ All specimens underwent analysis for tumor grade, and for ER, progesterone receptor (PR), and HER2 protein expression in a central lab as previously described^47^ (including HER2 assessment by 2007 ASCO-CAP guidelines used at the time^48^), and for Oncotype DX Recurrence Score in the Genomic Health, Inc. (Redwood City, CA) laboratory. The weighted distributions of the characteristics of the patients in the sample of 600 cases and the E2197 cases not in the sample are compared in Supplemental Table 3, indicating that the characteristics for the included vs. not-in-sample cohorts were similar except for race, where the weighted percent of whites is higher for those included (91.1%) compared with those not included (86.4%).

The parent E2197 clinical trial was approved by the institutional review boards of all participating institutions and was carried out in accordance with the Declaration of Helsinki, Food and Drug Administration Good Clinical Practices, and local ethical and legal requirements. The use of specimens for this project was approved the North American Intergroup Correlative Science Committee, by the MD Anderson Cancer Center Institutional Review Board (which oversees the ECOG-ACRIN Central Biorepository and Pathology Facility), and by the Albert Einstein College of Medicine Institutional Review Board (for the study principal investigator JAS).

### MetaSite *Breast*™ assay

The MetaSite *Breast*™ assay was for identifying and enumerating TMEM was performed in a commercial CLIA-certified laboratory (CLIA ID No. 22D2094085; MetaStat, Inc. Drydock Avenue, Boston MA), as previously described.^31^ Briefly, tissue sections (5 um formalin-fixed paraffin embedded on positively charged glass microscope slides) were derived from tumor blocks at the ECOG-ACRIN Central Biorepository and Pathology Facility (CBPF), sent to MetaStat, and upon receipt were dipped in paraffin and stored under non-oxidizing and non-hydrolyzing conditions until stained. Tissue samples were stained using a modified triple chromogen immunohistochemical stain for CD31-positive blood vessels using a rabbit anti-CD31 monoclonal antibody (AbCam/Epitomics Clone EP3095), for CD68-positive macrophages using an anti-CD68 mouse monoclonal antibody (Thermo Scientific Clone PGM1), and for Mena-positive tumor cells using an anti-Pan-Mena mouse monoclonal antibody (Gertler lab, MIT). CD31-positive blood vessels were visualized using blue chromogen, CD68-positive macrophages visualized using brown chromogen, and Mena using red chromogen. A pathologist reviewed each sample for quality and specific staining patterns based on established specificity criteria in addition to image capture of invasive cancer alone. Imaging was conducted using the Perkin Elmer Vectra 2 multispectral microscopy system (Perkin Elmer, Hopkinton, MA), which allows for spectral un-mixing of chromogens enabling highly accurate imaging and image analysis. For whole tissue imaging, up to 100 20X high-resolution images were acquired in areas of invasive tumor using established and validated image analysis algorithms. All images used in analysis were reviewed by trained pathologists for image quality and histologic specificity. TMEM identification and enumeration were accomplished through a combination of InForm (PerkinElmer) and VisioPharm (VisioPharm) image analysis software. InForm generates spectrally unmixed composite images representing spectrally pure chromogen channels for CD31, CD68, and Pan-Mena. These images were then used in a VisioPharm MetaSite identification algorithm where TMEM were identified as microanatomic structures that meet established criteria for direct contact of all three cells. The number of TMEM for each image were quantified and reported as MetaSite Score. MetaSite Score was defined as the sum of TMEM sites from each of the top three highest density 20X fields-of-view. Prior to evaluation of samples from the E2197 trial, the analytical precision, reproducibility, and accuracy of the fully automated MetaSite *Breast*™ clinical assay was demonstrated in an analytical validation study using formalin-fixed, paraffin-embedded (FFPE) tissue samples from patients with invasive breast cancer.^31^

### Statistical analyses

The primary endpoint for analysis was distant recurrence-free interval (DRFI), defined as time from entry on E2197 to first distant recurrence. DRFI is censored at the time last known to be free of distant recurrence. Because only the first recurrence of any type was recorded in the database, for patients with local or regional recurrence (before distant), DRFI is censored at the date of first recurrence. Other endpoints include recurrence-free interval (RFI), defined to be time from study entry to first recurrence of breast cancer at any site, censored at the date last known to be free of recurrence, and OS, defined to be the time from entry on E2197 to death from any cause, censored at the date last known to be alive. DRFI and RFI were defined in accordance with STEEP guidelines.^49^ The number of events for each subgroup, overall and by follow-up time, are shown in Supplemental Table 2. Because only 37 of 85 deaths (44%) in the HR+/HER2- group were due to breast cancer, we also evaluated breast cancer specific survival, defined as time from study entry until death due to breast cancer.

As previously described, although a biased sample was used, unbiased estimates of effect were obtained by weighting the contributions of observations by the inverse of the sampling fractions in the sampling groups (the strata by recurrence status combinations).^50^ While standard software can be used to compute the weighted estimates, special routines are often needed to compute correct standard errors. Distributions of the characteristics of patients were estimated using weighted proportions and weighted averages. Time to event distributions were estimated using weighted Kaplan–Meier estimators. Hazard ratios were estimated and regression analyses performed by fitting weighted partial likelihood models. All analyses were performed in R 3.2.3. Weighted partial likelihood models were fit using the coxph() function with weights. A locally written function was used to compute the corrected variance using the score residuals from the fitted model. For time-varying coefficient models, start and stop arguments are used to create data sets for the contribution at each event time. Evaluation of prognosis associated with categorical Recurrence Score included the classical definitions of low, intermediate, and high (<18, 18–30, >30), and also the definitions (<11, 11–25, >25) used in the Trial Assigning Individualized Options for Treatment (TAILORx).^29^ Based on the assumption that 621 specimens would be available (80% of original sample of 776 patients), it was estimated that the study would have 80% power using a two-sided 5% significance level to detect a hazard ratio of 2.0–2.2 associated with DRFI between the highest and the lowest tertile TMEM Score level, which was comparable to that observed in the original TMEM validation study (2.70, 95% CI: 1.39, 5.26).^26^ The study was conducted in accordance with reporting recommendations for tumor marker prognostic studies (REMARK) guidelines.^51^

### Data availability statement

The data sets generated during and/or analyzed during the current study are available in the NCTN/NCORP Data Archive (https://na01.safelinks.protection.outlook.com/?url=https%3A%2F%2Fnctn-data-archive.nci.nih.gov%2F&data=02%7C01%7CJSPARANO%40montefiore.org%7C882c9b32175548ab7eef08d4e7faffa1%7C9c01f0fd65e040c089a82dfd51e62025%7C0%7C0%7C636388512604292871&sdata=ACdv92L8ftF9KtBhkQLpCNh9zLPX3qhdDWN1WIp%2FH88%3D&reserved=0).

## Electronic supplementary material

Supplementary Tables 1–3

Supplementary Figures 1

Supplementary Figure 2
