# Supplementary material for: A metastasis biomarker (MetaSite Breast™ Score) is associated with distant recurrence in hormone receptor-positive, HER2-negative early-stage breast cancer
Source: NPJ Breast Cancer. 2017 Nov 8;3:42. doi: 10.1038/s41523-017-0043-5 (PMC5678158; doi:10.1038/s41523-017-0043-5)
Supplement: Supplementary file 1 — Supplementary Tables 1–3 [file 41523_2017_43_MOESM1_ESM.docx]

**Supplemental Table 1. P-values for Distant Recurrence. Breast Cancer Specific Survival, and Overall Survival for Continuous MetaSite Score Proportional Hazard and Time-Varying Coefficient Models**

|  |  | Time Varying Coefficient | |
| --- | --- | --- | --- |
| **Distant recurrence** | Proportional  Hazards | Overall  (2 df) | Nonproportionality |
| Overall | 0.88 | 0.002 | 0.003 |
| HR+/HER2- | 0.14 | 0.0002 | 0.01 |
| Triple negative | 0.27 | 0.31 | 0.21 |
| HER2+ | 0.37 | 0.16 | 0.07 |
| **Breast cancer specific survival** |  |  |  |
| Overall | 0.07 | 0.18 | 0.95 |
| HR+/HER2- | 0.04 | 0.06 | 0.54 |
| Triple negative | 0.99 | 0.33 | 0.14 |
| HER2+ | 0.80 | 0.50 | 0.34 |
| **Overall survival** |  |  |  |
| Overall | 0.85 | 0.48 | 0.23 |
| HR+/HER2- | 0.42 | 0.21 | 0.17 |
| Triple negative | 0.13 | 0.26 | 0.59 |
| HER2+ | 0.93 | 0.91 | 0.67 |

Proportional Hazards is from the test for an effect of continuous MetaSite score in the proportional hazards model using all follow-up. Time Varying Coefficient uses the model with the variables continuous MetaSite score and the product of Continuous MetaSite score and time. In this model, Overall is from the joint test of the two variables, which gives the test for any effect of MetaSite score at any follow up time, and Nonproportionality is from the test of whether the effect of the MetaSite score is changing with time.

**Supplemental Table 2. Distribution of Outcome Events by Breast Cancer Subtype and Time Period**

|  |  | Follow-up Time  (Years) | | |  |
| --- | --- | --- | --- | --- | --- |
| Subtype | Endpoint | 0-5 | 5-10 | >10 | Total |
| Triple negative | Recurrence | 45 | 3 | 3 | 51 |
|  | Distant Recurrence | 32 | 1 | 1 | 34 |
|  | Death (all) | 38 | 14 | 13 | 65 |
|  | Breast Cancer Death | 28 | 6 | 3 | 37 |
| HR+, HER2- | Recurrence | 43 | 15 | 12 | 70 |
|  | Distant Recurrence | 35 | 7 | 7 | 49 |
|  | Death (all) | 24 | 36 | 25 | 85 |
|  | Breast Cancer Death | 12 | 16 | 9 | 37 |
| HER2+ | Recurrence | 26 | 5 | 1 | 32 |
|  | Distant Recurrence | 15 | 4 | 0 | 19 |
|  | Death (all) | 17 | 11 | 2 | 30 |
|  | Breast Cancer Death | 14 | 6 | 0 | 20 |
| Combined | Recurrence | 114 | 23 | 16 | 153 |
|  | Distant Recurrence | 82 | 12 | 8 | 102 |
|  | Death (all) | 79 | 61 | 40 | 180 |
|  | Breast Cancer Death | 54 | 28 | 12 | 94 |

**Supplemental Table 3. Comparison of case-control sample to E2197 subjects not in sample**

|  | In Sample | | Not in Sample | |
| --- | --- | --- | --- | --- |
|  | # cases  (n=600) | weighted  % (SE) | # cases  (n=2352) | weighted  % (SE) |
| Arm AT | 310 | 50.0(0.9) | 1166 | 50.0(0.9) |
| Arm AC | 290 | 50.0(0.9) | 1186 | 50.0(0.9) |
| Age <=40 | 89 | 14.4(1.5) | 315 | 13.7(0.7) |
| 41-50 | 212 | 36.1(2.1) | 836 | 35.3(1.0) |
| 51-60 | 185 | 31.3(2.0) | 782 | 33.2(1.0) |
| >60 | 114 | 18.2(1.6) | 419 | 17.8(0.8) |
| Premeno | 282 | 47.3(2.2) | 1120 | 47.6(1.0) |
| Postmeno | 318 | 52.7(2.2) | 1232 | 52.4(1.0) |
| Race: White | 538 | 91.1(1.1) | 2036 | 86.4(0.7) |
| Hispanic | 6 | 0.9(0.4) | 60 | 2.6(0.3) |
| Black | 45 | 6.5(1.0) | 171 | 7.4(0.5) |
| Asian | 2 | 0.4(0.3) | 36 | 1.5(0.3) |
| Native American | 3 | 0.3(0.2) | 4 | 0.2(0.1) |
| Other/Unk | 6 | 0.7(0.3) | 45 | 1.9(0.3) |
| ER positive | 332 | 65.4(1.1) | 1584 | 64.9(0.9) |
| PR positive | 285 | 56.0(1.6) | 1394 | 57.0(0.9) |
| Tumor Size: <=2cm | 284 | 50.1(2.2) | 1279 | 53.9(1.0) |
| 2.1 to 5.0cm | 292 | 46.2(2.1) | 986 | 42.4(1.0) |
| > 5 cm | 24 | 3.7(0.8) | 81 | 3.4(0.4) |
| Nodes: 0 positive | 356 | 63.8(0.9) | 1533 | 64.0(0.9) |
| 1 positive | 144 | 21.6(1.3) | 489 | 21.5(0.8) |
| 2 positive | 69 | 10.5(1.1) | 211 | 9.3(0.6) |
| >=3 positive | 31 | 4.1(0.7) | 119 | 5.2(0.5) |
| Grade: Low | 49 | 9.0(1.3) | 259 | 10.6(0.6) |
| Intermediate | 197 | 37.5(2.0) | 935 | 38.9(1.0) |
| High | 326 | 48.6(2.0) | 1030 | 45.1(1.0) |
| Unk | 28 | 5.0(1.0) | 128 | 5.3(0.5) |
| HER2: Low | 272 | 45.0(2.2) | 1083 | 45.9(1.0) |
| High | 126 | 21.0(1.8) | 538 | 23.0(0.9) |
| Unk | 202 | 34.0(2.1) | 731 | 31.0(1.0) |

ER, PgR, Grade, and HER2 are per local institution determination (central ER, PR, grade and HER2 were not available for not-in-sample group); HER2 coding includes IHC 2+ and 3+ in the ‘High’ group.Patients with > 3 nodes positive were not eligible for E2197, but 7 were enrolled (3 with 4, 3 with 5, and 1 with 9), all in the not-in-sample group.
