# Supplementary material for: A metastasis biomarker (MetaSite Breast™ Score) is associated with distant recurrence in hormone receptor-positive, HER2-negative early-stage breast cancer
Source: NPJ Breast Cancer. 2017 Nov 8;3:42. doi: 10.1038/s41523-017-0043-5 (PMC5678158; doi:10.1038/s41523-017-0043-5)
Supplement: Supplementary file 2 — Supplementary Figures 1 [file 41523_2017_43_MOESM2_ESM.docx]

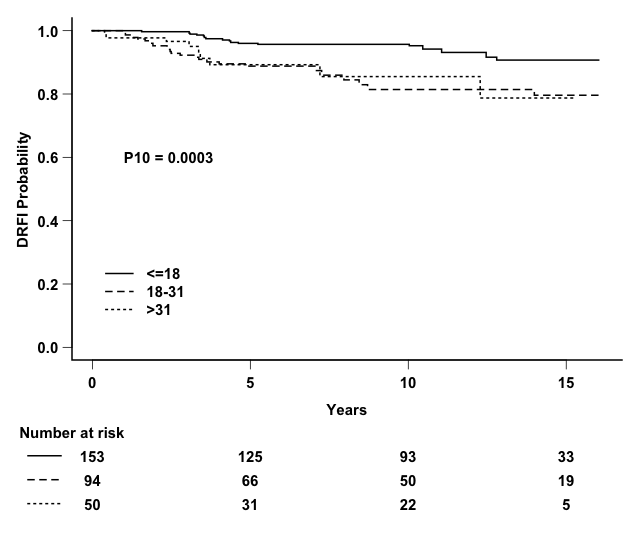


**Supplemental Figure 1A.** Distant relapse free interval (DRFI) by Oncotype Recurrence Score using classical definitions of low, intermediate, and high Recurrence Score (<18, 18-30, >30) in HR+/HER2- disease (P10: p-value computed truncating follow-up at 10 years).


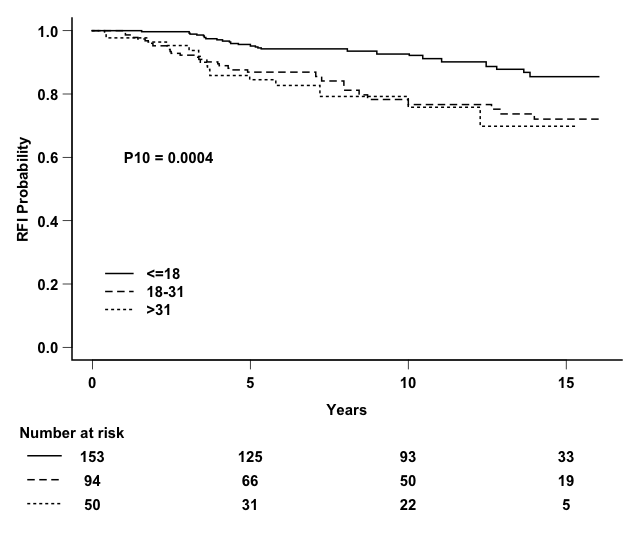


**Supplemental Figure 1B.** Relapse free interval (RFI) by Oncotype Recurrence Score using classical definitions of low, intermediate, and high Recurrence Score (<18, 18-30, >30) in HR+/HER2- disease (P10: p-value computed truncating follow-up at 10 years).


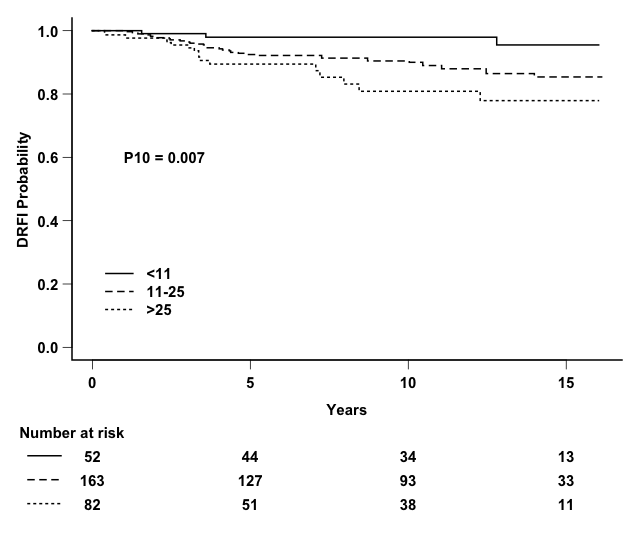
**Supplemental Figure 1C**. Distant relapse free interval (DRFI) by Oncotype Recurrence Score using TAILORx definitions of low, intermediate, and high Recurrence Score (<11, 11-25, >25) in HR+/HER2- disease (P10: p-value computed truncating follow-up at 10 years).


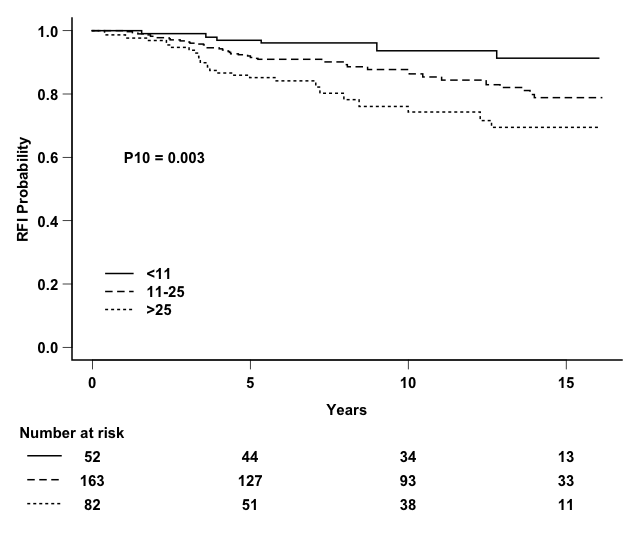


**Supplemental Figure 1D**. Relapse free interval (RFI) by Oncotype Recurrence Score using TAILORx definitions of low, intermediate, and high Recurrence Score (<11, 11-25, >25) in HR+/HER2- disease (P10: p-value computed truncating follow-up at 10 years).
