# Supplementary material for: A metastasis biomarker (MetaSite Breast™ Score) is associated with distant recurrence in hormone receptor-positive, HER2-negative early-stage breast cancer
Source: NPJ Breast Cancer. 2017 Nov 8;3:42. doi: 10.1038/s41523-017-0043-5 (PMC5678158; doi:10.1038/s41523-017-0043-5)
Supplement: Supplementary file 3 — Supplementary Figure 2 [file 41523_2017_43_MOESM3_ESM.docx]

**Supplemental Figure 2.** Consort diagram

Enrolled in trial E2197 (N=2952)

| Excluded | N=1373 |
| --- | --- |
| Ineligible (as of 10/29/2005) | 67 |
| Did not consent to future use of tissue | 341 |
| Unknown ER/PR status | 10 |
| Early lost to follow-up | 11 |
| No blocks submitted | 880 |
| No tumor in block | 64 |

Included in case:cohort study (n=1579)

| Recurrences / non-recurrences (as of May 23, 2006) | 191/1388 |
| --- | --- |

|  | Recurrence/Non-Recurrence |
| --- | --- |
| Exclusions (based on analyzability of the samples at Genomic Health): | N=56 |
| Insufficient tumor | 3/15 |
| Path Ineligible (on GHI review) | 3/10 |
| Insufficient RNA | 5/12 |
| QPCR Sample Quality | 1/6 |
| Average Reference Gene Count >35 | 1/0 |
| Total Exclusions | 13/43 |

Patients eligible for inclusion in cohort (N=1523)

Number selected for inclusion in previous case:cohort study (N=776)

| Recurrences / non-recurrences (as of May 23, 2006) | 178/598 |
| --- | --- |

Final cohort included in this analysis (N=600) current case:cohort study

- Recurrence (N=153)/Non-Recurrence (N=447) (as of May 27, 2016)

(n=620)

MetaSite Score Assay not performed (N=20)

- Insufficient tissue (N=3)
- Insufficient invasive tumor (N=3)
- Staining failure (N=1)
- Limitation to MetaSite assay (N=4)

Included in current case:cohort study (N=620)

Tissue depleted from prior analyses (N=156)
